# Fc effector functions in RNA viral infections: mechanisms of antiviral immunity and implications for vaccine design

**DOI:** 10.3389/fimmu.2026.1772257

**Published:** 2026-05-11

**Authors:** Jiale Zhang, Chuang Li, Yuanyuan Wu, Lili Wang, Jie Yu, Aofan Wang, Wenwen Kong, Mingzhe Ning, Jing Chen, Yuxin Chen

**Affiliations:** 1 Department of Laboratory Medicine, Nanjing Drum Tower Hospital Clinical College of Jiangsu University, Nanjing, China; 2 Department of Laboratory Medicine, Nanjing Drum Tower Hospital Clinical College of Nanjing University of Chinese Medicine, Nanjing, Jiangsu, China; 3 Department of Laboratory Medicine, Joint Institute of Nanjing Drum Tower Hospital for Life and Health, College of Life Science, Nanjing Normal University, Nanjing, Jiangsu, China; 4 Clinical Research Center, The Second Hospital of Nanjing, Nanjing University of Chinese Medicine, Nanjing, Jiangsu, China; 5 Department of Laboratory Medicine, Nanjing Drum Tower Hospital, Nanjing University Medical School, Nanjing, Jiangsu, China

**Keywords:** antibody-dependent cellular cytotoxicity, antibody-dependent enhancement, Fc engineering, Fc-mediated effector functions, Fcγ receptors, vaccine-induced immunity

## Abstract

Neutralizing antibodies (NAbs) have long been the principal correlate of antiviral protection. Evidence now indicates that antibody Fc-mediated effector functions play indispensable and context-dependent roles in antiviral immunity. Through interactions between the fragment crystallizable (Fc) domain and Fc receptors (FcRs) or complement components, antibodies mediate a broad spectrum of effector mechanisms, including antibody-dependent cellular cytotoxicity (ADCC), antibody-dependent cellular and neutrophil phagocytosis (ADCP and ADNP), and complement activation, contributing to viral control beyond direct neutralization. In this review, we integrate recent evidence on Fc effector biology across major viral infections, including severe acute respiratory syndrome coronavirus 2 (SARS-CoV-2), influenza virus, human immunodeficiency virus (HIV), Ebola virus (EBOV), and dengue virus (DENV). We discuss how Fc-FcR interactions shape antiviral immune outcomes, modulate vaccine efficacy, and influence the balance between protective immunity and immunopathology, including antibody-dependent enhancement (ADE). We focus on the experimental strategies used to assess Fc-mediated functions and on the inherent limitations of *in vitro* assays and animal models in defining their physiological relevance in humans. We explore how different vaccine platforms and immunization strategies shape Fc effector profiles, specifically through antibody subclass selection, Fc glycosylation patterns, and engagement with Fcγ receptors. We also summarize emerging approaches to Fc engineering and glycan modification that aim to enhance antibody efficacy while limiting adverse immune activation. This review summarizes current understanding of Fc effector functions in antiviral immunity and discusses their relevance for the design of next-generation vaccines and antibody-based therapies.

## 1 Introduction

RNA viruses, such as severe acute respiratory syndrome coronavirus 2 (SARS-CoV-2), influenza virus, human immunodeficiency virus (HIV), Ebola virus (EBOV), and dengue virus (DENV), pose a major global public health threat owing to their high mutation rates and rapid evolutionary capacity (1). Neutralizing antibodies (NAbs) exert antiviral activity by binding viral surface proteins through their fragment antigen-binding (Fab) regions, thereby blocking viral attachment, entry, or early stages of replication and serving as a primary barrier to infection (2). However, many RNA viruses have evolved diverse immune evasion strategies, and the epitope specificity of NAbs inherently limits their breadth of protection against rapidly emerging variants.

As a result, increasing attention has been directed toward the broader and more versatile fragment crystallizable (Fc)-mediated effector functions of antibodies. Much of the supporting evidence for Fc-mediated functions comes from *in vitro* systems and animal models (3), while the exact role of Fc in antiviral protection in humans remains unclear. Fc-mediated responses are typically evaluated through *in vitro* assays such as antibody-dependent cellular cytotoxicity (ADCC), antibody-dependent neutrophil phagocytosis (ADNP), antibody-dependent cellular phagocytosis (ADCP), complement-dependent cytotoxicity (CDC), and antibody-dependent enhancement (ADE).

Fc-mediated effector functions represent a complex immunological phenomenon that is difficult to fully recapitulate *in vitro*(4). In Fc receptor (FcR)-based assays, PBMCs used immediately after freeze-thaw or after resting show altered ADCC and NK cell activity compared with freshly isolated PBMCs (4). This methodological variability can affect both the magnitude and interpretation of Fc-dependent responses. As a result, careful interpretation and contextualization of *in vitro* findings are necessary.

Despite these limitations, natural infection or vaccination induces a diverse repertoire of antibodies with distinct specificities and effector functions (5). Antibody-mediated immune protection results from the coordinated actions of antibodies with diverse effector functions (6). These functions may act synergistically in some contexts but can also interact in complex or even antagonistic ways *in vivo*, indicating that neutralizing activity alone does not fully account for protective immunity (6). Continual vaccine updates are required to keep pace with viral evolution. This has prompted increasing interest in additional immune mechanisms capable of counteracting viral escape from NAbs. Fc effector functions enable antibodies not only to recognize viral antigens but also to recruit and activate immune components, thereby promoting pathogen clearance and elimination of infected cells (5). Distinct vaccine platforms can differentially shape antibody Fc effector profiles, influencing the quality of antiviral immunity beyond neutralization (7). In this context, this review focuses on several major pandemic-associated pathogens, including SARS-CoV-2, influenza virus, HIV, EBOV, and DENV, highlighting the protective roles of Fc effector functions and discussing their implications for vaccine design and therapeutic antibody development.

## 2 Fc receptor signaling and effector mechanisms in antiviral immunity

**Figure 1:**
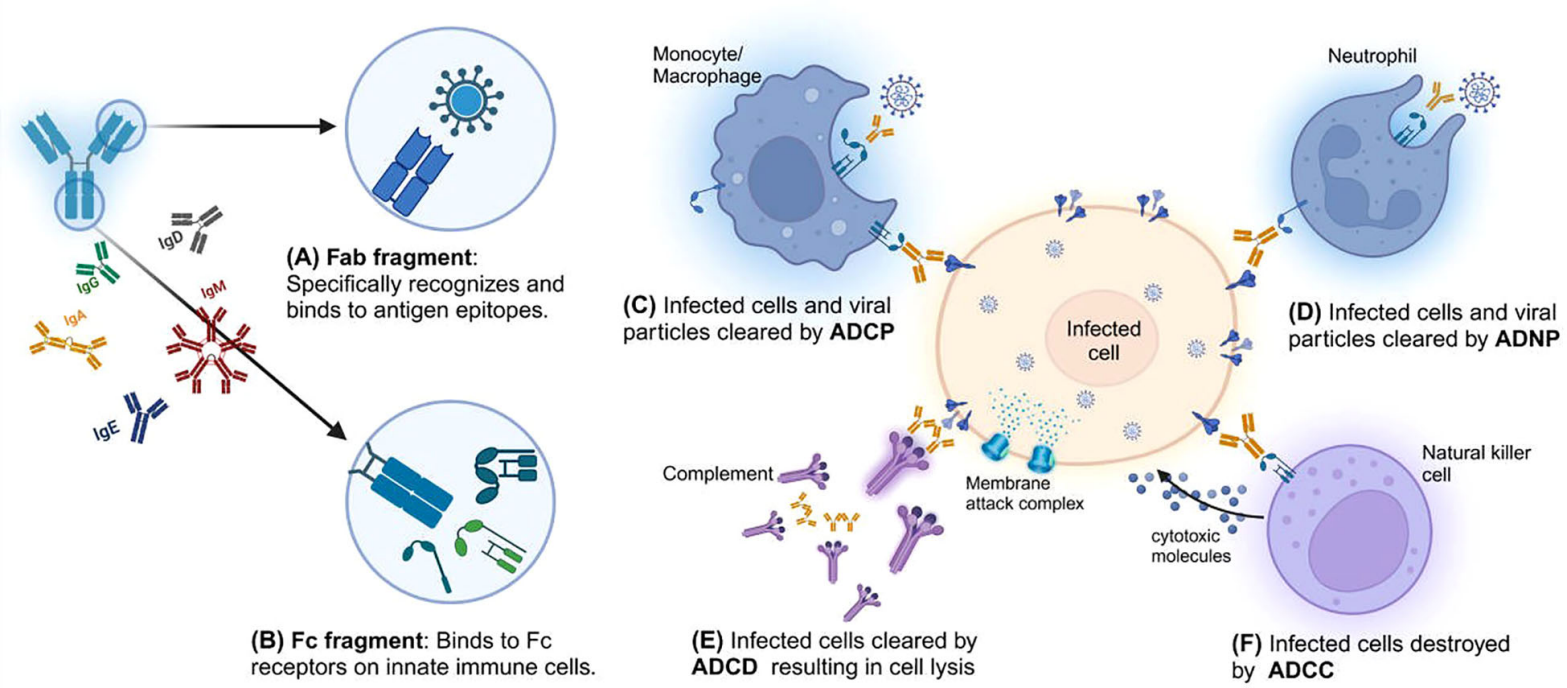
Mechanisms of Fc-mediated effector functions. **(A)** The Fab (Fragment antigen-binding) region of the antibody is responsible for antigen recognition and binds specifically to epitopes on pathogens or infected cells, providing antigen specificity. **(B)** The Fc (Fragment crystallizable) region of the antibody interacts with immune system receptors, such as Fc receptors (FcRs) and complement proteins, linking the adaptive and innate immune systems. **(C)** Antibody dependent cellular phagocytosis (ADCP): Monocytes and macrophages recognize and engulf antibody-opsonized infected cells or viral particles via FcRs, leading to their clearance. **(D)** Antibody dependent neutrophil phagocytosis (ADNP): Neutrophils recognize and phagocytose antibody-coated pathogens or infected cells, contributing to immune defense. **(E)** Antibody dependent complement deposition (ADCD): The Fc region activates the complement through the classical pathway, leading to membrane attack complex (MAC) formation and lysis of infected cells. **(F)** Antibody dependent cellular cytotoxicity (ADCC): Natural killer (NK) cells bind to the Fc region of antibodies attached to infected cells via FcγRIII (CD16), triggering cytotoxic molecule release to destroy infected cells.

Fc-mediated effector functions represent a central mechanism by which antibodies exert antiviral activity beyond neutralization (6). Antibody effector functions are mediated through interactions between the Fc fragment and Fc receptors (FcRs) expressed on immune cells, including monocytes, macrophages, dendritic cells (DCs), granulocytes, and natural killer (NK) cells, and through cooperation with the complement system (**Figure 1**) (8). These interactions enable antibodies to exert antiviral activities beyond neutralization and to act against diverse antigens, including viral structural proteins and glycoproteins (**Table 1**).

**Table 1:** Summary of viral targets and fc-mediated effector functions.

Virus	Target protein	Related Fc-mediated effector functions	References
SARS-CoV-2	Spike (S) protein	ADCP	(9)
		ADNP	(10)
		ADCC	(11)
		ADCD	(12)
	Nucleocapsid (N) protein	ADCC	(13)
		ADCP	(14)
		ADCP/ADCD	(15)
	Membrane (M) protein	ADCC	(16)
Influenza virus	Hemagglutinin (HA) stalk	ADCD	(17)
		ADCP	(18)
		ADCC	(19)
		ADNP	(20)
	Neuraminidase (NA)	ADCC	(21)
		ADCP	(22)
	Matrix Protein 2 ectodomain (M2e)	Not reported	Not reported
HIV	Env	ADCC	(23)
		ADCP	(24)
		ADCD	(25)
	p24	ADCP/ADCC	(26)
Ebola virus	Glycoprotein (GP)	ADCP/ADNKA/ADCD	(27)
		ADNP	(28)
	Soluble glycoprotein (sGP)	ADCP/ADNKA/ADCD/ADNP	(28)
Dengue virus	Envelope (E) protein	ADCP/ADNP/ADCD/ADCC	(29)
	Non-Structural Protein 1 (NS1)	ADCP/ADNP/ADCD/ADCC	(29)

This table summarizes the viral surface or structural proteins targeted by antibodies and the corresponding Fc-mediated effector functions reported to be associated with these targets for each virus. ADCP, antibody-dependent cellular phagocytosis; ADNP, antibody-dependent neutrophil phagocytosis; ADCC, antibody-dependent cellular cytotoxicity; ADCD, antibody-dependent complement deposition; ADNKA, antibody-dependent natural killer cell activation.

Fc receptors (FcRs) comprise Fcγ receptors (FcγRs), Fcα receptors (FcαRs), Fcϵ receptors (FcϵRs), Fcμ receptors (FcμRs), and the neonatal Fc receptor (FcRn).Among these, FcγRs bind IgG subclasses with differential affinities, with IgG3 exhibiting the strongest binding, followed by IgG1, IgG4, and IgG2 (30). FcγRI is a high-affinity receptor capable of binding monomeric IgG, whereas members of the FcγRII and FcγRIII families are low-affinity receptors that primarily recognize IgG within immune complexes through avidity-dependent interactions (31). Genetic polymorphisms in FcγRs can alter receptor-IgG binding properties. For instance, the FcγRIIIa V158F polymorphism affects binding to IgG1 and IgG3, while the FcγRIIa H131R polymorphism modifies the interaction with IgG2 (32). In addition to its role in IgG transport and recycling, FcRn contributes to antigen presentation and the regulation of humoral immunity (33). Other Fc receptors, including FcϵRI, FcαRI, and FcμR, mediate immune responses associated with IgE, IgA, and IgM, respectively (34–36).

Innate immune cells typically co-express multiple activating FcγRs (FcγRI [CD64], FcγRIIa [CD32a], FcγRIIc [CD32c], and FcγRIIIa [CD16a]) together with the inhibitory FcγRIIb (CD32b), thereby enabling the fine-tuned integration of activating and inhibitory signals and contributing to immune homeostasis (32). Neutrophils predominantly express FcγRIIIb, with lower levels of FcγRIIa (37). Macrophages express a broader repertoire of FcγRs, including FcγRI, FcγRIIa, FcγRIIIa, and the inhibitory FcγRIIb, which together support efficient phagocytosis and immune complex clearance (32). NK cells predominantly express FcγRIIIa as their principal activating FcγRs (8). Dendritic cells (DCs) co-express both activating and inhibitory FcγRs, particularly FcγRIIa and FcγRIIb, enabling fine regulation of immune activation and antigen presentation (8). In contrast, B cells primarily express the inhibitory receptor FcγRIIb, which mediates negative feedback regulation of B cell receptor (BCR) signaling (8). Upon engagement by immune complexes, activating FcγRs cluster on the cell surface and initiate signaling through immunoreceptor tyrosine-based activation motifs (ITAMs), whereas inhibitory FcγRs transmit signals via immunoreceptor tyrosine-based inhibitory motifs (ITIMs) (32). Phosphorylation of ITIM motifs promotes the recruitment of inhibitory phosphatases, including SHP-1, SHP-2, and SHIP-1, which counteract activating ITAM-mediated signaling pathways and thereby maintain immune homeostasis (38). In contrast, engagement of activating FcγRs induces Src family kinase–mediated phosphorylation of ITAMs, which leads to the recruitment and activation of the tyrosine kinase Syk. Activated Syk subsequently triggers multiple downstream signaling cascades that are partly cell type-dependent. These pathways include the PI3K–Akt signaling pathway, which contributes to cell survival, metabolic regulation, and cytoskeletal remodeling, as well as the PLCγ–Ca^2+^–PKC axis, which promotes intracellular Ca^2+^ mobilization and activates Ras-dependent MAP kinase signaling pathways, including the MEK–ERK cascade (32, 39). These Fc-FcR signaling networks enable a broad spectrum of antibody-mediated outcomes, including ADE and effector functions such as ADCC and ADCP (40–42).

The complement system works alongside FcγR-mediated pathways. Fc fragment binding to C1q triggers the classical complement cascade, leading to ADCD (43). Complement fragments such as C3b and iC3b act as potent opsonins and cooperate with FcR signaling to drive efficient phagocytosis and pathogen clearance (44). Engagement of complement receptors CR1 and CR2 enhances antigen presentation and supports B cell memory formation, linking innate and adaptive immunity (45). Fc-mediated functions help clear viruses, but excessive FcγR activation can also promote immunopathology by releasing inflammatory cytokines. Antibody isotype, subclass, and Fc glycosylation further shape these effector responses. Afucosylation enhances FcγRIIIa binding, whereas sialylation is typically linked to anti-inflammatory activity (**Table 2**) (49, 50).

**Table 2:** N-glycosylation on IgG Fc receptor binding and their relevance in RNA viruses.

Fc Glycosylation ^a^	Glycosylation type	Impact on FcγR/C1q binding	Effect on Fc effector functions	Associated infectious diseases	References
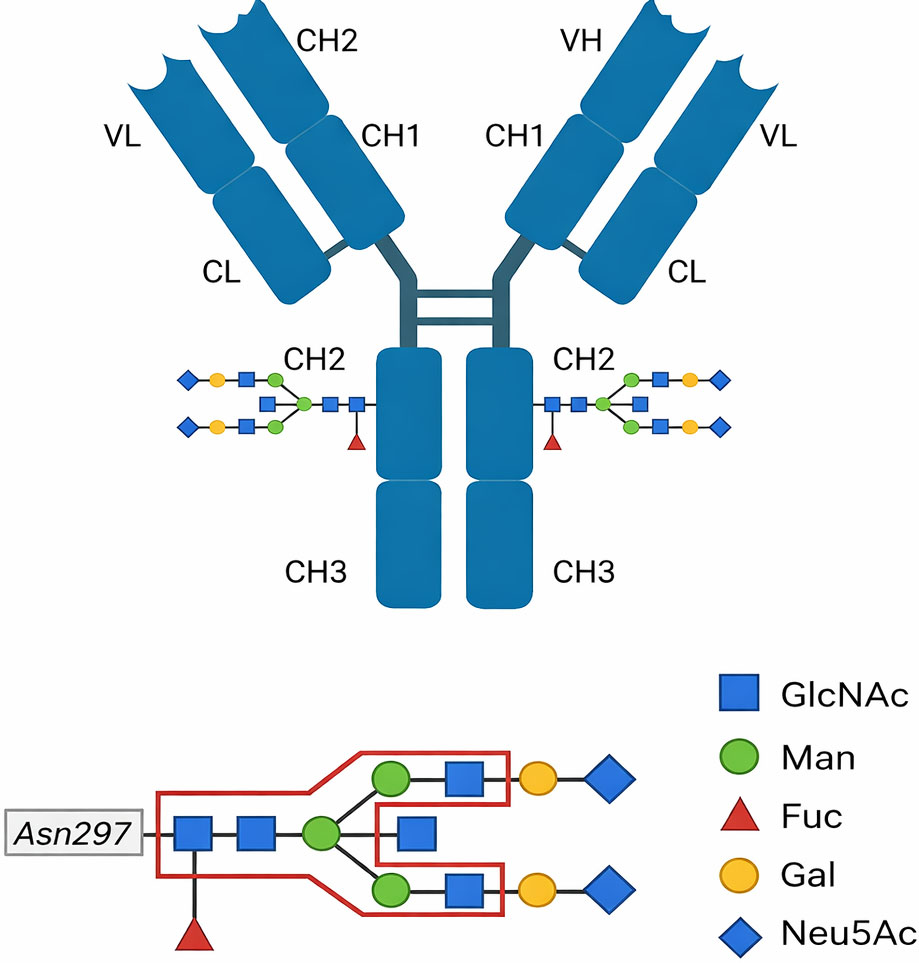	Afucosylation (Absence of Core Fucose)	↑Enhances FcγRIIIa binding	↑ Enhances ADCC	SARS-CoV-2	(46)
				HIV	(47)
				Ebola virus	(48)
	Sialylation (Addition of Neu5Ac)	↑ May enhance FcγRIIb binding; ↓ May reduce FcγRIIIa and C1q binding	↓ May suppress ADCC and CDC	SARS-CoV-2	(49)
				HIV	
				Influenza virus	
	Galactosylation (Addition of Gal)	↑ May enhance C1q, FcγRIIIa and FcγRIIa binding	↑ May enhance CDC, ADCC and ADCP	HIV	(49)
				SARS-CoV-2	
				Ebola virus	

^a^ IgG Fc glycans at Asparagine 297 (Asn 297) in the CH2 domain are complex biantennary N-glycans. The core Fc glycan consists of four N-acetylglucosamine (GlcNAc) and three mannose (Man) residues, which can undergo modifications such as core fucosylation (Fuc), bisecting GlcNAc, and galactose (Gal) addition. In the presence of galactose, N-acetylneuraminic acid (NeuAc, sialic acid) may also be incorporated. The glycosylation pattern of IgG plays a crucial role in modulating Fc-mediated immune functions, impacting interactions with Fc receptors (FcRs), complement activation, and effector functions.

## 3 Balancing protection and enhancement: Fc effector functions in RNA virus infection

Fc-mediated immunity represents a double-edged sword in viral infections, mediating protective antiviral functions while also carrying the potential to drive ADE (40). This balance between protection and immunopathology varies across viral pathogens. In DENV infection, ADE is well established, particularly during secondary heterotypic infections, where pre-existing non-neutralizing antibodies enhance viral entry and disease severity via FcγR-mediated pathways (40, 51). In contrast, for SARS-CoV-2, HIV, and Ebola virus, ADE immunopathological mechanisms have primarily been demonstrated *in vitro*, while definitive clinical evidence in humans remains limited (40, 52). Advances in Fc engineering further highlight the possibility of rationally modulating Fc-FcγR interactions to enhance therapeutic efficacy while minimizing immunopathology. The following sections discuss the protective and pathogenic roles of Fc-mediated immunity in SARS-CoV-2, DENV, EBOV, HIV, and influenza virus infections.

### 3.1 Fc effector functions in SARS-CoV-2 infection: complementary roles beyond neutralization

Since the outbreak of SARS-CoV-2 in 2019, natural infections have resulted in over 780 million people being infected globally, with more than 7.1 million deaths, posing a severe threat to public health (53). SARS-CoV-2 is an enveloped, positive-sense single-stranded RNA virus characterized by a surface spike (S) glycoprotein composed of two subunits: S1 and S2 (54). The S1 subunit mediates attachment to the host angiotensin-converting enzyme 2 (ACE2) receptor, whereas the S2 subunit facilitates the fusion of the viral envelope with the host cell membrane (55). NAbs target the S1 subunit, particularly the receptor-binding domain (RBD) and, to a lesser extent, the N-terminal domain (NTD), to block viral entry by preventing interaction with the ACE2 receptor, inhibiting subsequent infection and dissemination (56).

SARS-CoV-2 undergoes frequent mutation in the S protein (57). Although these mutations may variably reduce neutralizing activity, Fc-mediated effector functions complement neutralization by providing broader functional immune protection that remains active against escape variants (58–60). Evidence from both clinical and experimental studies supports a role for Fc activity in disease outcomes. For example, Fc responses directed against the S protein have been associated with improved survival in hospitalized patients (61, 62). Beyond natural infection, vaccines across multiple platforms elicit robust Fc-mediated effector functions with broad cross-reactivity, which may contribute to sustained protection even when neutralizing activity declines. mRNA vaccines such as BNT162b2 and mRNA-1273 induce rapid humoral responses characterized by high levels of S- and RBD-specific IgG, broad FcγR engagement, and coordinated Fc effector activities, including ADCC and ADCD (63). Nevertheless, a direct causal relationship between *in vitro* Fc functional readouts and clinical protection in humans has yet to be established. Fc-mediated effector functions decline over several months following vaccination across platforms, including mRNA and inactivated vaccines, particularly against antigenically divergent variants such as Omicron. Booster immunization can rapidly restore multiple Fc functions, including ADNP, and partially reshape Fc profiles, potentially contributing to broader and more durable protection (64). However, the long-term durability of Fc responses across vaccine platforms remains incompletely defined. Comparative analyses further reveal qualitative differences in Fc responses across vaccine platforms. Adenoviral vector vaccines such as Ad26.COV2.S have been reported to induce lower peak neutralizing antibody titers compared with mRNA vaccination regimens (65). However, systems serology analyses suggest that adenoviral vector vaccination may elicit enhanced FcγRIIIa binding and ADCP activity in certain contexts, despite lower neutralizing titers (66). In non-human primate models, protein subunit vaccines such as NVX-CoV2373 formulated with Matrix-M™ induce focused antibody responses, with the adjuvant playing a key role in shaping Fc effector profiles by promoting IgG subclasses with enhanced FcγR binding (67). Repeated mRNA vaccination may qualitatively reshape antibody subclass composition. Recent studies have reported that, in adults, repeated mRNA immunization is associated with a decline in ADCP and ADCD activity, correlating with increased levels of S-specific IgG4 (68). An elevated anti-S IgG4/IgG1 ratio has been shown to inversely correlate with NK cell activation capacity and ADCD activity. A similar trend has been observed in elderly populations, where repeated booster immunization is accompanied by increased IgG4/IgG1 ratios and relatively reduced antibody-mediated NK cell activation when normalized to virus-specific IgG levels (69). However, current evidence remains largely correlative, and direct mechanistic studies establishing a causal link between IgG4 subclass shifts and altered antibody-dependent natural killer cell activation (ADNKA) are still lacking (69).

Fc-mediated immune functions are regulated by multiple factors, including FcγR genetic polymorphisms, IgG subclass composition, and memory B-cell responses. The extensive genetic diversity of FcγRs influences IgG binding affinity and downstream signaling, contributing to interindividual variability in immune responses and therapeutic efficacy (42, 70). For example, the rs1801274 polymorphism in FcγRIIa has been associated with the risk of death in COVID-19 patients (71). IgG subclasses further contribute to this functional heterogeneity. IgG4 binds weakly to FcγRIIIa and may limit ADCC activity, yet retains the ability to mediate moderate ADCP via FcγRI and FcγRIIa, suggesting a potential role in fine-tuning immune responses rather than acting solely as an inhibitory isotype (43). Overall, FcγR genetic diversity and IgG subclass–specific functional patterns underscore the complexity of Fc-dependent immunity (70). Beyond antibody neutralization, Fc-dependent mechanisms can enhance antiviral immunity by promoting FcγR-mediated antigen cross-presentation and CD8^+^ T-cell responses (72, 73). Consistently, studies of therapeutic antibodies against SARS-CoV-2 demonstrate that intact Fc effector functions are required for optimal protection. In K18-hACE2 transgenic mouse and hamster models, treatment with COV2-2050 markedly reduced inflammatory pathology, with CD8^+^ T cells identified as key contributors to antibody-mediated efficacy. FcγR engagement primarily modulated immune responses to limit immunopathology and reduce viral burden rather than directly promoting viral clearance (74). However, substantial differences in Fc structure and FcγR expression between animals and humans limit direct extrapolation of these mechanisms to human infection. High-resolution analyses further indicate that Fc-mediated protection can operate independently of neutralization. Distinct B-cell germlines, including IGHV1-46, IGHJ6-1, IGHV2-5, and IGHJ4-1, retain strong ADCP and ADCD activity against SARS-CoV-2 variants despite reduced neutralizing capacity (75). Moreover, antibodies with higher levels of somatic hypermutation exhibit enhanced Fc functionality, highlighting the contribution of B-cell maturation to the breadth and strength of Fc-dependent immune responses (76).

Fc-FcR interactions play a context-dependent dual role in SARS-CoV-2 infection, promoting antiviral immunity under balanced immune activation while driving inflammatory pathology when signaling is excessive or dysregulated. Fc-dependent signaling enhances antiviral defense through the activation of innate immune effector cells. NK cells eliminate virus-infected cells through FcγRIIIa-mediated ADCC (77). Elevated FcγRIIIa-mediated activation and afucosylated IgG responses have been associated with severe COVID-19 and the significant contribution of the FcγRIIIa-158-V/F polymorphism to antibody-mediated NK cell activation against SARS-CoV-2 LALA (78, 79). Fc-dependent mechanisms also extend to neutrophil extracellular trap formation (NETosis). Both SARS-CoV-2 virions and immune complexes present in patient sera have been shown to activate NETosis, particularly through IgA-mediated immune complexes engaging FcαR signaling (80). However, under dysregulated or excessive immune activation, Fc-dependent signaling may contribute to adverse outcomes. These include ADE of infection, FcR-driven inflammatory amplification, and tissue damage associated with uncontrolled NETosis. *In vitro* studies using FcγRIIb-high cell line models have shown that SARS-CoV-2 monoclonal antibodies such as MW01 and MW05 can induce ADE, with stronger effects observed in Raji cells than in Daudi cells (81, 82). Beyond facilitating viral entry, FcγR-mediated signaling can amplify inflammatory responses independently of enhanced infection. FcγRIIIa-dependent activation may exert either protective or pathological effects depending on the magnitude and timing of signaling. Afucosylation of spike-specific IgG enhances FcγRIIIa engagement and amplifies immune activation in NK cells, monocytes, macrophages, and neutrophils. While such enhanced signaling may contribute to viral control, excessive activation has been associated with heightened inflammation in severe COVID-19 and may contribute to immunopathology, including acute respiratory distress syndrome (ARDS) (46). Notably, whereas afucosylation enhances FcγRIIIa engagement and has been linked to hyperinflammation in severe COVID-19, the IgG4 subclass exhibits reduced activating FcγR affinity. These opposing qualitative shifts further underscore that Fc structural remodeling may either amplify or attenuate inflammatory signaling, depending on context. Beyond ADE, although early Fc-mediated neutrophil activation and NETosis may facilitate viral containment, excessive or sustained NETosis can drive tissue damage and inflammatory pathology (83, 84). Elevated neutrophil activation markers and IgA-associated NETosis have been observed in severe COVID-19 patients, linking dysregulated Fc-dependent responses to disease severity (85, 86).

Current Fc engineering strategies increasingly focus on mitigating the risk of ADE while preserving or enhancing antiviral efficacy. For instance, MW05 LALA mutations in the Fc region reduce FcγRs engagement and have been shown to confer protection in rhesus macaque models of SARS-CoV-2 infection by suppressing viral replication, reducing viral loads, and alleviating pulmonary inflammation without exacerbating tissue damage (81). Conversely, selective enhancement of FcγRs engagement can also improve therapeutic efficacy, as exemplified by the GAALIE Fc variant, which demonstrates potent antiviral activity at reduced doses in Syrian hamster models. The reduced C1q binding of this variant suggests that FcγRs-mediated mechanisms, rather than complement activation, dominate its protective effects (87). Fc engineering approaches have also been extended to ACE2-Fc decoy proteins, where distinct Fc modifications fine-tune antiviral mechanisms. Specific Fc mutations differentially regulate FcγR engagement and complement activation: for instance, the H429Y mutation disrupts FcγR interactions while enhancing neutralization, whereas the H429F mutation promotes binding to the terminal complement complex C5b-C9, converting ACE2-Fc into a potent mediator of complement-dependent cytotoxicity. In addition, Fc glycosylation modifications can enhance FcγRIIIa-mediated immune activation, highlighting the combined roles of Fc structural and glycan regulation in shaping ACE2-Fc functionality (88). In the K18-hACE2 mouse model, ACE2-Fc exhibits high efficacy against the replicative Omicron variant, providing broad neutralization of SARS-CoV-2 variants of concern while activating innate immune cells via its Fc domain to facilitate clearance of free virus particles and infected cells (89). The therapeutic potential of Fc-engineered antibodies and decoy proteins, but the risk of ADE associated with Fc manipulation remains a subject of ongoing debate.

### 3.2 Fc effector functions in influenza virus infection: mechanisms of broadly protective antibody activity and their regulatory implications

Influenza viruses remain a major concern for global infectious disease surveillance and vaccine development, causing approximately one billion seasonal cases annually and up to 650, 000 deaths globally (90). They are enveloped, negative-sense, single-stranded RNA viruses with segmented genomes and are classified into types A, B, C, and D. Among viral surface antigens, haemagglutinin (HA) and neuraminidase (NA) exhibit the greatest antigenic variability (91). Current influenza vaccines predominantly elicit NAbs against the highly variable head region of the HA glycoprotein, which is the principal driver of antigenic drift (91, 92). Continual antigenic drift and occasional antigenic shift underscore the need for vaccines capable of providing broad and durable protection (93).

In contrast to the variable HA head, the conserved HA stalk region represents a central target for broadly protective antibodies and universal vaccine strategies. Antibodies directed against this region primarily mediate protection through Fc-dependent mechanisms, including ADCC, NETosis, and phagocytosis (94, 95). Passive transfer studies demonstrated that the HA stalk-specific antibody FI6 conferred complete protection in mice and ferrets, whereas protection was substantially reduced in the absence of FcγR engagement. Similarly, broadly Nabs targeting conserved HA and NA epitopes require FcγR interactions for effective protection against lethal influenza infection (94). The importance of Fc effector functions extends to therapeutic antibodies. The monoclonal antibody 3C08, which targets the NA head base, showed minimal *in vitro* antiviral activity yet effectively protected mice, whereas Fc modification to a LALA variant markedly increased viral loads, highlighting the necessity of Fc-mediated interactions (96). In H7N9-infected mice, ADCP was identified as a dominant protective mechanism, with HA stalk–specific IgG and IgA inducing neutrophil activation via Fc receptor engagement (97). Consistently, depletion of alveolar macrophages significantly diminished the protective efficacy of HA stalk-targeting antibodies (98). Fc-mediated immunity also contributes to influenza prevention at the population level. Pre-pandemic samples from children prior to the 2009 H1N1 outbreak revealed higher ADCC activity and elevated FcγRIIIa/FcγRIIa-binding antibodies in uninfected individuals (99). ADCC and ADCP activities were likewise detected in unexposed adults and increased with age, suggesting the gradual accumulation of cross-reactive Fc-mediated immunity shaped by prior influenza exposure. Host immune factors, including age, infection history, and original antigenic sin, further modulate these responses.

Vaccination studies similarly demonstrate the induction of Fc-dependent antibody functions. In adults and children vaccinated with inactivated subunit vaccines (ISV) or live-attenuated influenza vaccines (LAIV), ADCC-mediating antibodies are commonly induced and correlate with reduced viral replication and milder clinical symptoms (100). However, the frequent use of CD16–GFP NK cell lines may not fully reflect the functional capacity of primary human NK cells. Comparative studies in non-human primates further show that most booster strategies, except live-attenuated vaccines, elicit substantial ADCC responses (100). Notably, RhAd52-H1/WI22 and mRNA-H1/WI22 boosters induce multiple Fc-mediated functions, including ADCD, ADCP, and ADNP, while robust Fc activity in bronchoalveolar lavage fluid is observed primarily following intratracheal boosting, highlighting the influence of immunization route on mucosal Fc functionality (101). In parallel, mRNA-based influenza vaccines such as mRNA-1010 induce stronger FcR-binding antibodies, faster maturation of functional humoral responses, broader HA-specific antibody repertoires, and enhanced NK cell and neutrophil activation compared with adjuvanted inactivated vaccines (102). Vaccine platform and administration route substantially shape Fc effector profiles, with important implications for influenza vaccine efficacy and design.

Protection by Fc effector functions requires tight regulation to avoid immunopathology. Antigenic imprinting can influence the quality and durability of Fc-mediated responses by shaping clonal selection and epitope targeting (18). In animal models, vaccines or monoclonal antibodies preferentially inducing Fc-dependent responses in the absence of sufficient neutralization have been associated with exacerbated pulmonary pathology (103, 104). Evidence from rational antigen engineering demonstrates that immunodominance can be overcome and humoral responses redirected toward subdominant yet broadly protective epitopes, providing a conceptual framework for universal influenza vaccine design (105). Precisely optimizing Fc effector activity in antibody engineering and vaccination strategies is needed to maximize protection while minimizing tissue damage (106).

### 3.3 Fc effector functions in HIV infection: distinct Fc “fingerprints” and their association with protective immunity

Over the past four decades, the global HIV epidemic has been driven primarily by two strains: HIV-1 and HIV-2. HIV-1 remains the predominant strain worldwide, causing an estimated 84 million infections and 40 million deaths globally, whereas HIV-2 is largely restricted to regions such as West Africa and India (106). HIV particles are positive-sense single-stranded RNA viruses enveloped by a host-derived lipid bilayer containing the envelope glycoprotein complex (Env), composed mainly of gp120 and gp41 (107, 108). gp120 represents the primary target of neutralizing antibodies but is highly prone to antigenic drift, limiting the effectiveness of conventional neutralization-based strategies (107).

Although antiretroviral therapy (ART) has markedly improved survival, long-term treated individuals, particularly older patients, remain at increased risk of metabolic and cardiovascular comorbidities, reflecting both treatment-related effects and persistent immune dysfunction (109–111). In addition, latent viral reservoirs in resting CD4^+^ T cells enable viral rebound following treatment interruption (112). In this context, broadly neutralizing antibodies (bNAbs) have emerged as complementary interventions. *In vitro* studies demonstrate that bNAbs mediate ADCC in cell culture systems and eliminate HIV-1 infected lymphocytes through engagement of NK cells (113). Preclinical and clinical studies increasingly associate Fc effector activity with improved viral control and reduced disease severity (114).

Systems serology analyses across multiple HIV vaccine trials have revealed distinct Fc “fingerprints” associated with differential efficacy outcomes. In the RV144 trial, the only regimen to demonstrate modest protective efficacy, V1V2-specific IgG3 responses were inversely correlated with infection risk and formed a coordinated Fc effector network characterized by IgG1- and IgG3-mediated ADCC and ADCP (115). IgG3 responses closely tracked IgG1 functional activity, suggesting that subclass distribution may serve as a surrogate marker of coordinated Fc-mediated immunity (115). In contrast, the VAX003 regimen predominantly elicited gp120-specific IgG4 responses and displayed limited coordination among Fc effector functions, a profile observed in the absence of protective efficacy (115). These comparative analyses suggest that qualitative differences in Fc network organization, rather than antibody magnitude alone, may distinguish protective from non-protective responses. More recent vaccine platforms further support this concept. The IPCAVD001 and Ad26-based regimens induced more integrated Fc-mediated immune networks dominated by IgG1 responses and associated with ADCC and ADCP activity (115). In rhesus macaques, Ad26/Env vaccination elicited robust Fc effector functions, with ADCP, ADCD, and ADCC associated with protective efficacy against SIV or SHIV challenge (116). Similarly, mosaic Ad26/Ad26+gp140 regimens were robustly immunogenic in both humans and non-human primates and conferred substantial protection against SHIV-SF162P3 challenge in monkeys, with both Fc-mediated antibody responses and T cell immunity correlating with efficacy (117). Even in trials that failed to demonstrate overall efficacy, *post hoc* immune analyses provided insight into Fc-dependent mechanisms. In the DNA/rAd5 (HVTN 505) trial, ADCP activity was inversely associated with infection risk, and FcγRIIa-dependent antibody responses were implicated in modulation of susceptibility (24, 118). In ART-treated individuals receiving heterologous Ad26/MVA vaccination, enhanced ADCP responses were associated with delayed viral rebound (119, 120). Although debate remains regarding the precise mechanistic contribution of Fc effector functions, particularly in RV144, these studies collectively underscore the importance of coordinated Fc-mediated immune networks as potential determinants of HIV vaccine efficacy (121–123).

Despite their potential protective roles, Fc effector functions in HIV infection may also carry inherent risks if dysregulated. Early vaccine trials, including AIDSVAX (VAX004), raised concerns regarding possible immune-mediated enhancement, as individuals with low Env-specific antibody responses exhibited higher infection rates compared with placebo recipients (124). Definitive evidence for classical ADE in HIV remains lacking. Nevertheless, these observations underscore the complexity of Fc-mediated immunity. A similar pattern emerged in the RV144 trial, where vaccine-induced Env-specific IgA responses were positively correlated with infection risk (122), potentially through interference with protective IgG-mediated effector functions, highlighting the context-dependent and double-edged nature of Fc activity. Precise modulation, rather than indiscriminate amplification, of Fc engagement is therefore required. In this regard, Fc engineering provides a rational strategy to optimize antibody effector functions while minimizing unintended immune activation. Rational Fc modifications can fine-tune antibody-FcγR interactions, enhancing immune cell–mediated clearance of infected cells while limiting uncontrolled Fc signaling. For example, afucosylated antibodies exhibit enhanced binding particularly to FcγRIIIa, resulting in amplified NK cell–mediated cytotoxicity (125). Building on this principle, Fc-engineered variants incorporating afucosylation or GASDALIE mutations further augment NK cell activation and antiviral activity in preclinical models (125). Rationally optimized Fc functions thus offer substantial therapeutic potential in HIV antibody-based interventions.

### 3.4 Fc effector functions in EBOV infection: non-neutralizing antibody cocktails may confer unexpected protection against EBOV via Fc effector functions

EBOV is a highly lethal negative-strand RNA virus transmitted through bodily fluids, causing Ebola virus disease (EVD) with an overall case fatality rate of approximately 40–50% (126, 127). Although primarily endemic to Africa, global mobility increases the risk of international spread (127). Early antibody studies suggested that neutralization alone may be insufficient for protection. The neutralizing antibody KZ52, one of the first EBOV-specific antibodies identified with high affinity for the viral glycoprotein (GP), conferred complete protection in guinea pigs but failed to protect non-human primates (NHPs) from lethal EBOV challenge, underscoring the limitations of neutralization-centric paradigms (128, 129). In contrast, immune profiling of EVD survivors has revealed durable multifunctional Fc-mediated antibody responses. While neutralizing titers induced by the rVSV-ZEBOV vaccine may decline over time, survivors maintain robust Fc-dependent responses for up to 24 months and can retain immune memory for more than 15 years. Multiple studies demonstrate that sera from EBOV survivors exhibit IgG-mediated Fc-dependent antiviral activity, particularly ADCC mediated through FcγRIIIa engagement (130, 131). In addition, EBOV survivor sera contain IgA antibodies capable of mediating ADNP, highlighting the contribution of multiple isotypes and Fc-dependent pathways to durable immunity (130).

NAbs are not the sole mechanism of protection against EBOV, as Fc effector functions provide an additional pathway to prevent infection. In NHP models, Ad26/Env vaccination elicited robust Fc-mediated responses, and ADCP, ADCD, and ADCC were identified as correlates of protection (132). ZMapp comprises the non-neutralizing antibody c13C6 (derived from MB-003) together with the neutralizing antibodies c2G4 and c4G7 (derived from ZMAb), and collectively exhibits pronounced Fc-mediated effector functions. Although all EBOV monoclonal antibodies mediate comparable levels of ADCP through FcγRIIa, significant differences are observed between neutralizing and non-neutralizing antibodies in ADNP, suggesting differential Fc interactions with FcγRIIIb (133, 134). mAb114 also retains Fc-mediated effector functions and significantly improves survival in NHPs (135, 136). *In vitro* ADCC activity may contribute to the protective efficacy of mAb114 against lethal EVD in rhesus macaques (135). In guinea pig models, antibodies engineered to signal exclusively through the FcγRIIIa pathway (REGN3471), as well as antibodies retaining both neutralizing and Fc effector functions (REGN3470), demonstrated *in vivo* therapeutic efficacy when administered as monotherapy, further supporting a role for Fc-mediated mechanisms in protection against EBOV (136). Additionally, *in vitro* analyses revealed that KL-2G12 and KL-1F11 exhibited the highest activities in ADCC reporter assays, whereas KL-2G12 and KL-1F8 showed the strongest activity in ADCP assays (137). Further evidence was provided by the Viral Hemorrhagic Fever Immunotherapeutic Consortium (VIC). Their analysis of mAbs demonstrated that antibodies with strong neutralizing activity but limited *in vivo* protection elicited weak NK cell activation, whereas those with weaker neutralization yet effective *in vivo* protection induced robust NK cell and phagocytic responses (129). Another study in mouse models confirmed that ADCC, rather than neutralization, was the primary protective mechanism (138). Moreover, studies in infected mouse models have demonstrated that engineered antibody variants, such as FTEA and KWES, can induce high levels of complement activation. While these variants showed moderate induction of NK cell–mediated effector activity and monocyte phagocytosis, their ability to robustly engage the complement system highlights an important antiviral mechanism (139, 140). Consistent with this notion, independent studies have shown that GP-specific monoclonal antibodies against EBOV can induce CDC by promoting C3 deposition on GP-expressing cells (139). Collectively, these findings converge on the concept that the Fc domain is indispensable for coordinating immune effector mechanisms against EBOV infection, with direct implications for the rational design of vaccines and therapeutic antibodies.

Fc effector functions play a significant role in EBOV infection, but the underlying mechanisms remain incompletely understood. Classic pathways such as ADCP, ADCD, and ADNP have been explored, yet several questions persist. These include how Fc engages FcRs, how FcR polymorphisms influence immunity, and whether neutrophils act through neutrophil extracellular traps (NETs). Antibody glycosylation critically determines the strength and type of Fc functions. Expression systems vary in the glycosylation profiles they produce, which may have variable or even adverse effects on therapeutic efficacy. Therefore, designing therapeutic antibodies requires attention to both the regulatory role of glycosylation and its potential downsides. Preliminary evidence in Ebola virus survivors suggests that specific Fc effector functions may associate with long-term sequelae. In particular, ADCP, ADCD, and ADNKA were inversely correlated with the risk of hearing impairment, while higher ADNP responses correlated with increased joint pain (27). Fc effector functions may contribute to immune regulation independently of neutralizing antibody levels in Ebola viral infection. Future studies should prioritize these unresolved questions to fully harness Fc-mediated immunity against EBOV.

### 3.5 Fc effector functions in DENV infection: anti-DENV IgG antibodies can mediate both protective immunity and immunopathology through Fc receptor engagement

DENV is a Flavivirus transmitted by *Aedes* mosquitoes, which causes illnesses ranging from mild dengue fever to severe dengue hemorrhagic fever (DHF) and dengue shock syndrome (DSS). According to the WHO, dengue is the most prevalent arthropod-borne viral infection, putting an estimated 3.9 billion people at risk, with more than 7.6 million cases reported as of April 2024 (141). DENV is an enveloped, positive-sense, single-stranded RNA virus of the Flaviviridae family (142). The viral genome encodes a single open reading frame (ORF) that is processed into three structural proteins (C, prM/M, E) and seven non-structural proteins (NS1, NS2A, NS2B, NS3, NS4A/2K, NS4B, and NS5) (142). Protective immunity against DENV has traditionally been attributed to Nabs targeting the E protein, which block viral attachment and fusion via Fab-dependent mechanisms (143). However, the efficacy of NAbs is limited by serotype differences, and during secondary infection, cross-reactive antibodies often fail to neutralize heterologous serotypes, instead promoting viral entry via FcγRIIa and FcγRIIIa, a phenomenon known as ADE (144–146). This process is concentration dependent, with FcγRIIb acting as a potential negative regulator (147, 148). By contrast, NS1 represents another critical immunological target. Anti-NS1 antibodies can bind membrane-associated NS1 (mNS1) on infected cells and mediate clearance through Fc-dependent mechanisms such as ADCP and complement activation. However, excessive soluble NS1 (sNS1) competes for antibody binding and impairs complement activity, diminishing protective efficacy (149, 150).

Fc effector functions in DENV infection are both crucial and complex (151). In a cohort of Nicaraguan children previously infected with DENV, pre-existing total IgG antibodies with Fc effector functions, including ADCP, ADCD, and ADNKA mediated by DENV3 E-specific antibodies, were associated with protection against secondary DENV3 infection (132, 133). Polyfunctional antibodies targeting both E and NS1 induced ADCD, facilitating opsonization and clearance of viral particles and infected cells, and correlated with asymptomatic infections. Higher levels of DENV3 E-specific ADCD and ADCP were linked to the absence of hemorrhagic symptoms (29). *In vitro* studies show that antibodies can trigger ADCC against DENV-infected cells, though responses vary across individuals due to genetic polymorphisms in FcγRs and differences in antibody glycosylation (152). Higher ADCC activity before or early during secondary infection correlated with lower viremia in DENV3 infection. However, Fc-mediated functions may also contribute to disease severity. One important example is the role of Fc glycosylation in modulating FcR interactions. DENV infection has been associated with increased levels of afucosylated IgG1 (153). This glycoform exhibits a higher affinity for the activating receptor FcγRIIIa, and the level of afucosylated IgG1 serves as a predictive marker for dengue disease severity (153). Experimental studies also show that the pathogenic effects of anti-DENV antibodies can be mediated through FcγRIIIa-dependent activation of splenic macrophages, resulting in inflammatory sequelae and increased mortality in mouse models (154). Standard *in vitro* measurements of Fc-mediated functions, including ADE assays, may not fully capture the complexity of Fc-dependent immune responses occurring *in vivo*, as they often lack the cellular diversity, dynamic signaling feedback, and tissue-specific microenvironment that shape immune outcomes in the host (155). Dengue-Zika virus cross-reactive antibodies can mediate complement-dependent viral lysis and have been associated with protection against severe dengue, further underscoring the diverse outcomes of Fc-mediated antibody responses (156). These findings point to the dual nature of Fc-dependent immunity in dengue: antibody traits like glycosylation patterns, epitope specificity, and receptor engagement decide whether Fc functions protect or harm.

Given the central role of ADE and the context-dependent effects of Fc receptor engagement, Fc engineering has emerged as a promising strategy to improve the safety profile of dengue therapeutic antibodies. *In vitro* studies show that Fc-modified human monoclonal antibodies, including LALA-B3B9 and N297Q-B3B9, effectively inhibited ADE mediated by human anti-DENV sera in K562 cells (157). These Fc-engineered antibodies retained broad neutralizing activity against all four dengue virus serotypes and did not promote viral infection. Consistent with these findings, Fc-modified 3G9 antibodies exhibited more rapid viral clearance in DENV-2-infected mouse models. The reduced ADE observed in the engineered variants may contribute to their improved *in vivo* efficacy; however, direct validation of this mechanism remains limited by the lack of animal studies employing well-established ADE models (158).

## 4 Methods for assessing Fc-mediated effector functions

Current knowledge of Fc-mediated antibody effector functions is shaped by the experimental systems used, and methodological differences contribute to variability across studies. Existing approaches can be broadly classified into *in vitro* functional assays, system-level analyses, and *in vivo* models, which differ substantially in mechanistic resolution and translational relevance.

FcγR reporter assays typically employ engineered cell lines expressing specific human FcγRs coupled to reporter genes, such as Jurkat/FcγRIIa/NFAT-Luc or Jurkat/FcγRIIIa/NFAT-Luc cells, to quantitatively assess FcγR activation (159). These assays are highly standardized, quantitative, and receptor-specific, enabling effective comparison of different FcγR subtypes and polymorphic variants (160). However, these systems primarily measure receptor signaling strength rather than the execution of biological effector functions by authentic immune cells, as they do not include primary effector cell populations. Consequently, FcγR reporter assays are best suited for mechanistic dissection of Fc-FcγR interactions and comparative evaluation of Fc variants, rather than for direct inference of antibody effector functions *in vivo*.

ADCC and ADCP assays provide functional readouts of Fc-mediated effector activity by incorporating immune effector cells. ADCC assays are typically based on co-culture systems in which antibody-opsonized target cells are incubated with effector cells, including primary immune cells such as peripheral blood mononuclear cells (PBMCs) or primary human NK cells, engineered NK cell lines, or, in some studies, Jurkat reporter cell lines expressing FcγRIIIa as surrogate models to assess FcγR-mediated signaling activation (161). However, these reporter systems do not recapitulate the execution of cytotoxic effector functions. ADCC assay formats vary widely in experimental design and functional readouts. These readouts may be based on target cell-associated metrics (e.g., cell lysis or viability) or effector cell-associated metrics (e.g., degranulation or cytotoxic molecule release), which limits direct comparability across studies (162). In contrast, ADCP assays typically employ phagocytic cell systems, such as the THP-1 monocytic cell line, to evaluate antibody-dependent phagocytosis mediated predominantly by FcγRIIa, providing insights into the potential role of antibody Fc functions in antigen clearance and immune activation (163). Phagocytic activity measured *in vitro* does not necessarily predict *in vivo* protection, as FcγR-mediated phagocytes not only engulf and eliminate pathogens but also process and present antigens to shape downstream adaptive immunity (e.g., T cell priming and B cell activation) (164). Despite these methodological limitations, ADCC and ADCP assays remain highly relevant in studies of infectious diseases and vaccination and are widely used to assess Fc-mediated effector functions of therapeutic or vaccine-induced antibodies.

To strengthen causal inference at the mechanistic level of antibody effector functions, blocking studies are widely used to assess whether antibody activity depends on Fc–FcγR interactions (165). Common approaches include the use of FcγR-specific blocking antibodies, Fc-engineered antibodies with reduced or abolished FcγR binding capacity, as well as genetic manipulation of FcγRs (e.g., receptor deficiency or functional attenuation) to assess the contribution of FcγRs (165, 166). These strategies help distinguish Fc-dependent from Fc-independent effects and support causal interpretation of FcγR-mediated mechanisms. However, conclusions from blocking studies can be influenced by several factors, including incomplete receptor blockade, off-target effects, and variability across experimental systems and biological contexts (42, 167).

With the advancement of high-throughput technologies, transcriptomic analyses and systems serology approaches are increasingly used to examine Fc-related immune features and their associations with immune outcomes at the systems level. Transcriptomic approaches, based on bulk or single-cell RNA sequencing, enable the identification of immune pathways and cellular programs associated with Fc-mediated responses and help define Fc-driven immune activation states (168). Similarly, systems serology integrates multidimensional Fc features, including FcR binding properties, glycosylation patterns, and functional readouts, to characterize Fc-mediated immunity across multiple parameters (115). These approaches have been widely applied in vaccine studies and human cohorts to identify Fc-related correlates of protection. Nevertheless, systems serology analyses also rely heavily on statistical associations and model-dependent interpretations, and therefore cannot, on their own, establish causal relationships between Fc features and immune outcomes (169). Accordingly, system-level approaches are best viewed as complementary tools that contextualize Fc effector functions, rather than as substitutes for direct functional assays.

Finally, *in vivo* animal models, including FcγR-humanized mice as well as non-human primates and other animal systems, provide an indispensable physiological context for evaluating the contribution of Fc-mediated effector functions to protection or pathology within integrated immune responses. These models enable assessment of Fc-dependent mechanisms in the setting of intact immune networks and have been widely used to validate Fc-driven effects in infection and therapeutic studies. However, pronounced species-specific differences in the Fc-FcγR axis, including receptor composition, expression patterns, and binding affinities, substantially limit the direct translation of findings from animal models to human immunity (42). The potential efficacy of Fc-mediated activities should be interpreted in an integrated manner that accounts for the experimental system employed, the level of evidence provided, and the specific biological context under investigation (**Table 3**).

**Table 3:** Fc effector function assays in virology.

Experimental methods	Fc effector function assessed	Experimental system	Strengths	Limitations	Applications	References
Fcγ Reporter Assays	FcγR activation and signaling potency	Fcγ-expressing reporter cell lines (e.g., Jurkat/FcγRIIa/NFAT-Luc or Jurkat/FcγRIIIa/NFAT-Luc cells expressing specific FcγRs coupled to reporter genes)	Quantitative comparison of FcγR subtypes and polymorphic variants; Suitable for screening Fc-engineered antibodies	Do not involve primary effector cells; Reporter signaling does not directly equate to biological effector functions	Mechanistic dissection of Fc–FcγR interactions; Fc engineering and antibody optimization	(159, 160)
ADCC Assays	Antibody-dependent cellular cytotoxicity	Primary human NK cells or PBMCs; Engineered NK cell lines; Jurkat reporter cell lines expressing FcγRIIIa	Direct functional readout of cytotoxic activity; Detecting antibody structure changes (antibody glycosylation or amino acid sequence);	High donor variability with primary cells; Assay conditions vary widely across studies	Evaluation of Fc-mediated cytotoxicity of therapeutic or vaccine-induced antibodies	(161, 162)
ADCP Assay	Antibody-dependent cellular phagocytosis	THP-1 cell lines	Reflects FcγRIIa-mediated effector functions; Links Fc activity to antigen clearance and immune activation	*In vitro* phagocytosis may not fully predict *in vivo* clearance efficiency	Assessment of antibody-mediated phagocytic activity in infection and vaccination studies	(163)
Blocking Studies	Fc–FcγR dependency of antibody functions	Receptor-blocking antibodies, Fc-mutant antibodies, Genetic receptor disruption	Strengthens causal inference regarding FcγR involvement	Incomplete or off-target blockade; Results depend on blocking efficiency and experimental context	Mechanistic validation of FcγR-mediated effects	(165–167)
Transcriptomic Approaches	Immune pathways and cellular programs associated with Fc-mediated responses	Bulk or single-cell RNA sequencing of immune cells	Provides system-level insight into Fc-driven immune activation states	Largely correlative; does not directly measure Fc effector functions	Characterization of immune signatures linked to Fc activity	(168)
Systems Serology Approaches	Multidimensional Fc features and their association with immune outcomes	Integrated analysis of Fc binding, glycosylation, and functional assays	Captures the complexity of Fc-mediated immunity across multiple parameters	Relies on statistical associations; interpretation depends on model selection	Identification of Fc correlates of protection in vaccine and cohort studies	(115, 169)
Animal Models	*In vivo* contribution of Fc effector functions to protection or pathology	Mouse models (including FcγR-humanized mice) and Non-Human Primate or other animal systems	Provide physiological context and integrated immune responses	Species-specific Fc–FcγR differences limit direct translation to humans	Validation of Fc-dependent mechanisms in infection and therapeutic studies	(42)

## 5 Conclusion and future work

Fc-mediated antibody effector functions act in concert with neutralizing antibodies to promote viral clearance and eliminate infected cells. Fc-dependent mechanisms can be protective but may also contribute to disease, depending on the context. These pathways contribute substantially to protection against infections such as EBOV and SARS-CoV-2, whereas FcR engagement during DENV infection can exacerbate disease severity through ADE, posing a major challenge for vaccine development. Evidence from the HIV RV144 trial suggests a potential protective contribution of Fc-mediated functions, although this remains a topic of debate. The importance of Fc effector functions in RNA virus infections may be associated with several intrinsic features of these viruses. High mutation rates facilitate escape from neutralizing antibodies, increasing reliance on Fc-mediated mechanisms as an additional layer of immune protection. RNA viruses also appear more prone to inducing ADE, likely due to their ability to exploit FcγR-mediated entry into immune cells. Infections such as SARS-CoV-2 and DENV are associated with pro-inflammatory Fc profiles, including increased afucosylated IgG, which enhances FcγRIIIa engagement and may contribute to disease severity. Together, these features suggest a conserved yet context-dependent contribution of Fc effector functions to immunity against RNA viruses. In contrast, Fc-mediated mechanisms are generally less central in many non-RNA virus infections, where viral latency, immune evasion strategies, and neutralizing antibody responses are often more critical for long-term control. The magnitude and outcome of FcR interactions are impacted by multiple factors, including antibody subclass, glycosylation profile, FcR polymorphisms, and host immune status. The lack of standardized functional assays and harmonized evaluation frameworks limits cross-study comparability and constrains the clinical utility of Fc functionality as a biomarker of immune protection. Structural Fc modifications, such as LALA substitutions that reduce FcγR binding, have been shown to mitigate ADE risk in certain experimental settings. In contrast, afucosylation enhances FcγRIIIa engagement and can potentiate ADCC and inflammatory activity. These opposing effects underscore the importance of context-specific Fc engineering. Such strategies provide a basis for developing next-generation antibody therapeutics with improved safety and effectiveness, and may also inform vaccine design by promoting antibodies with optimized Fc effector profiles that enhance protection while limiting pathogenic responses.

Methodological limitations also remain significant. Current *in vitro* models are unable to fully recapitulate the complexity and dynamic interplay of immune interactions observed *in vivo*, while animal models, including FcγR-humanized systems, only incompletely capture the characteristics of human Fc-FcR binding, thereby limiting direct translational relevance. Fc glycosylation profiling remains constrained by the lack of high-throughput and standardized analytical platforms, and data consistency across laboratories continues to be a challenge (170).

At the mechanistic level, further investigation is required to delineate the specific signaling pathways governing Fc-mediated effector functions. In neutrophils, in particular, the precise intracellular signaling cascades downstream of Fc receptor engagement remain incompletely defined (37). In addition, it is essential to elucidate how T-cell help and B-cell differentiation programs shape antibody Fc structure and glycosylation profiles, thereby indirectly modulating Fc-dependent immune functions. To comprehensively characterize context-dependent alterations in Fc activity across diverse viral infections, integrated multi-omics approaches combined with systems immunology modeling may provide novel insights into the underlying regulatory networks.

At the clinical level, prospective studies are needed to systematically evaluate the role of Fc-mediated functions in vaccine-induced protection, infection recovery, and antibody-based therapies. Such efforts should be supported by standardized functional evaluation frameworks and computational or AI-assisted modeling of Fc-FcR interactions to improve reproducibility and enable quantitative cross-study comparison. Importantly, emerging *in vivo* evidence indicates that Fc-FcγR-dependent effector functions contribute to vaccine-mediated protection against antigenically shifted SARS-CoV-2 variants (171). Recent studies further suggest that vaccine-elicited Fc activity is influenced by host FcγR genetics. Notably, repeated SARS-CoV-2 mRNA vaccination–associated increases in spike-specific IgG4 have been reported to modulate FcγR binding and downstream ADCC and ADCP in a polymorphism- and variant-dependent manner, highlighting the potential value of genotype-informed booster strategies, adjuvant selection, and antigen design (70). Concurrently, Fc-optimized “universal” vaccine concepts aim to induce broadly protective Fc effector profiles that remain effective despite antigenic drift. Consistent with this concept, systems serology studies have linked FcR–binding signatures and Fc effector activity with reduced susceptibility to influenza pandemic infection (99). These advances position Fc functionality as a critical bridge between immunological mechanisms and translational application, enabling vaccines and antibody therapeutics that combine neutralization with Fc-mediated breadth, durability, and safety across genetically and antigenically diverse populations.
